# Rhaponticin Blocks Glycolysis‐Mediated Histone Lactylation to Suppress Tongue Squamous Cell Carcinoma via HIF‐1α Activity Inhibition

**DOI:** 10.1002/kjm2.70254

**Published:** 2026-06-18

**Authors:** Yuan Wu, Liang‐Liang Yao, Lin Jiang, Zhi‐Yi Fang, Jia‐Jun Zhu, Yi‐Sen Shao, Wen‐Juan Wang, Xiao‐Wen Wan

**Affiliations:** ^1^ Jiangxi University of Chinese Medicine Nanchang Jiangxi China; ^2^ Department of Oral and Maxillofacial Surgery Affiliated Hospital of Jiangxi University of Chinese Medicine Nanchang Jiangxi China; ^3^ Internal Medicine of Traditional Chinese Medicine Affiliated Hospital of Jiangxi University of Chinese Medicine Nanchang Jiangxi China

**Keywords:** cisplatin, HIF‐1α, Lactylation, Rhaponticin, tongue squamous cell carcinoma

## Abstract

Tongue squamous cell carcinoma (TSCC) often develops therapeutic resistance. Metabolic reprogramming, particularly the glycolysis/lactate/histone lactylation axis, is a critical driver of tumor progression and therapy resistance. This study investigated the anti‐tumor mechanism of Rhaponticin (Rha), focusing on the metabolic/epigenetic axis involving hypoxia‐inducible factor 1α (HIF‐1α), glycolysis, and histone H3K18 lactylation (H3K18la). SCC9 and SCC9‐CisR cells were treated with Rha alone or in combination with 2‐deoxy‐D‐glucose (2‐DG) or sodium lactate (LacNa). The effects of Rha on glycolysis and H3K18la were evaluated by extracellular acidification rate assays, glucose/lactate quantification, and western blotting. Gain‐ and loss‐of‐function studies targeting HIF‐1α were conducted to determine the mechanistic dependency of Rha‐mediated effects. Subcutaneous and liver metastasis xenograft models were established for in vivo validation. Rha significantly suppressed glycolysis, glucose consumption, and lactate production, concomitant with the downregulation of HK2, LDHA, GLUT1, and H3K18la levels. Rha not only mimicked the effects of 2‐DG by inhibiting clonogenicity, migration, and invasion and decreasing cisplatin resistance in SCC9 or SCC9‐CisR cells but also reversed LacNa‐mediated promotion of these parameters, indicating that Rha acts through the glycolysis/lactate pathway. Rha inhibited tumor growth and liver metastasis, enhanced cisplatin sensitivity, and decreased H3K18la levels in mouse models. Mechanistically, Rha inhibited HIF‐1α, thereby attenuating glycolytic flux and decreasing lactate‐driven H3K18la. Rha inhibits TSCC progression and enhances cisplatin sensitivity by targeting HIF‐1α and repressing the glycolysis/lactate/H3K18la axis, suggesting that Rha is a promising candidate for disrupting metabolic/epigenetic crosstalk in TSCC.

## 1 Introduction

Among head and neck squamous cell carcinomas (HNSCCs), tongue squamous cell carcinoma (TSCC) is one of the most aggressive subtypes and is associated with poor prognosis [1]. Its global incidence is increasing markedly, with evidence suggesting that increasingly younger populations are being affected [2]. Despite ongoing improvements in multimodal therapy, primarily involving surgery combined with chemoradiation and targeted agents, the management of advanced TSCC remains a major challenge [3]. This difficulty stems mainly from high local recurrence, a marked propensity for metastasis, and treatment resistance, especially to first‐line drugs such as cisplatin (Cis) [3, 4, 5]. Therefore, elucidating the molecular mechanisms that drive malignant progression and therapeutic resistance in TSCC is crucial for discovering innovative drugs that can improve clinical outcomes.

A key driver of TSCC malignancy is tumor metabolic reprogramming, primarily through the Warburg effect, which is a fundamental feature of tumor metabolism [6, 7]. As a central metabolic hub, the Warburg effect drives multiple malignant processes, including tumor proliferation, invasion and metastasis, immune escape, angiogenesis, stemness maintenance, and chemotherapy resistance [8, 9]. Emerging evidence indicates that lactate produced via glycolysis acts as a key molecular link between metabolic dysregulation and malignant phenotypes by directly modulating gene transcription through histone lactylation, a newly identified epigenetic modification, thereby remodeling the tumor microenvironment [10]. Research has demonstrated that RRAS2, induced by H3 lysine 18 lactylation (H3K18la), is associated with adverse clinical outcomes in HNSCC and contributes to immune infiltration, chemotherapy resistance, and tumor invasion [11]. However, the specific mechanisms through which the glycolysis/lactate/histone lactylation axis promotes TSCC progression remain poorly understood.

Hypoxia‐inducible factor 1α (HIF‐1α) serves as a master switch that initiates and sustains the Warburg effect and the vicious cycle of lactate metabolism [12]. The hypoxic microenvironment stabilizes HIF‐1α, enabling it to induce the glycolytic program genes, including glucose transporters (GLUTs), hexokinase‐II (HK2), lactate dehydrogenase A (LDHA), and monocarboxylate transporter 4 (MCT4) [13]. Among these, MCT4 serves as an essential lactate‐H^+^ cotransporter, supporting sustained high‐rate glycolysis by effectively clearing lactate and protons [14]. Rhaponticin (Rha), a compound isolated from the root of *Rheum undulatum L*., exhibits anti‐tumor activity among its diverse pharmacological effects [15]. Our previous study demonstrated that Rha suppresses cancer cell stemness and enhances sensitivity to Cis in TSCC by targeting the HIF‐1α/MCT4 axis to inhibit Wnt/β‐catenin signaling [16]. However, whether Rha inhibits TSCC progression by targeting HIF‐1α to interfere with lactate‐induced histone lactylation has yet to be clarified.

This study investigated whether Rha suppresses the malignant biological behaviors of TSCC by regulating HIF‐1α‐mediated glycolysis‐driven H3K18la, thereby elucidating a novel mechanism by which Rha regulates metabolic‐epigenetic crosstalk in TSCC.

## 2 Materials and Methods

### 2.1 Cell Culture and Rha Treatment

Short tandem repeat‐authenticated SCC9 cells (catalog number CL‐0571; Procell, Wuhan, China) were maintained in DMEM/F12 (catalog number PM150312; Procell) supplemented with 1% penicillin–streptomycin (catalog number PB180120; Procell), 400 ng/mL hydrocortisone (catalog number 50–23‐7; Invivochem, Guangzhou, China), and 10% fetal bovine serum (FBS; catalog number 10100; Thermo, Waltham, MA, USA). Normal human oral epithelial cells (catalog number ABC‐TC4365; AcceGen Biotech, Fairfield, NJ, USA) were cultured using the Human Epithelial Cell Growth Medium Kit. All cultures were maintained at 37°C in a humidified atmosphere containing 5% CO_2_.

Following a previously described protocol [17], stable SCC9‐CisR cells were generated by subjecting parental SCC9 cells to progressively increasing concentrations of cisplatin (Cis; catalog number P4394; Sigma, St. Louis, MO, USA), ranging from 10^−7^ to 10^−6^ mol/L.

For Rha treatment, cells were exposed to 10 μM Rha (catalog number 155–58‐8; purity: > 99%; Abmole, Shanghai, China) for 24 h, with an equal volume of dimethyl sulfoxide used as the vehicle control. For treatment with 2‐deoxy‐D‐glucose (2‐DG; catalog number S4701; Selleck, Shanghai, China) or sodium lactate (LacNa; catalog number 71718; Sigma), parental SCC9 cells and their Cis‐resistant counterparts were divided into two groups: one received the compound alone (5 mM), and the other received the compound in combination with Rha.

### 2.2 Cell Transfection

Two specific siRNAs against HIF‐1α (sequences: #1, 5ˊ‐TGGTTACTCAGCACTTTTAGATG‐3′; #2, 5ˊ‐CACTTTTAGATGCTGTTTATAAT‐3′), along with a negative control siRNA (si‐NC), were commercially synthesized (Sangon, Shanghai, China). HIF‐1α overexpression was achieved by transfecting cells with the HIF‐1α vector (catalog number SR300295; OriGene, MD, USA), with the corresponding empty vector serving as the transfection control. All transfections were performed using the RiboFECT CP Transfection Kit (Ribobio, China) according to the manufacturer's protocol.

### 2.3 Seahorse Analysis

The extracellular acidification rate (ECAR) was assessed using the Seahorse XF Glycolysis Stress Test Kit (catalog number 103020–100; Agilent Technologies, Santa Clara, CA, USA). Before the assay, SCC9 and/or SCC9‐CisR cells (5 × 10^4^) were seeded into 96‐well plates and treated with Rha for 24 h. During the assay, which was performed on the Seahorse XF Analyzer (Agilent Technologies), glucose (10 mM), oligomycin (1 μM), and 2‐DG (50 mM) were sequentially injected. Data were analyzed using Seahorse Wave software.

### 2.4 Glucose Uptake and Lactate Production

Glucose consumption was assessed using a glucose uptake colorimetric assay kit (catalog number MAK083; Sigma), while lactate production was quantified using a lactate assay kit (catalog number K607‐100; BioVision, Milpitas, CA, USA). Both assays were performed according to the respective manufacturers' instructions.

### 2.5 Colony Formation Assay

SCC9 cells were seeded at 1 × 10^3^ cells per well in 6‐well plates and cultured for 10 days. The resulting colonies were fixed with 4% paraformaldehyde (catalog number P0099; Beyotime, Shanghai, China) for 15 min, stained with 0.5% crystal violet (catalog number 60506ES60; Yeasen, Shanghai, China) for 15 min, and photographed under an Olympus microscope (Tokyo, Japan). Colonies were counted by analyzing the images with ImageJ software (NIH, Bethesda, MD, USA).

### 2.6 Wound‐Healing Assay

A scratch was introduced into an SCC9 cell monolayer at 90% confluence in a 6‐well plate using a sterile 200‐μL pipette tip. To remove debris, the cells were washed twice with PBS and then cultured in FBS‐free medium. Scratch wounds were imaged at 0 h (baseline) and after 24 h of incubation.

### 2.7 Transwell Invasion Assay

To evaluate invasive capacity, cells were seeded into 8‐μm pore Transwell inserts pre‐coated with Matrigel (catalog number 354480; Corning, New York, USA). Medium containing 10% FBS was added to the lower compartment, and a suspension of SCC9 cells (1 × 10^4^) in 200 μL was plated in the upper chamber. After 24 h of incubation, invasive cells located on the lower surface of the membrane were fixed, stained with 0.5% crystal violet, and examined under an Olympus microscope. Cells were counted across five randomly selected microscopic fields.

### 2.8 Cell Viability Assay

SCC9 or SCC9‐CisR cells were seeded in 96‐well plates at 1 × 10^3^ cells per well and treated with a gradient of Cis concentrations (0, 2, 4, 8, 16, and 32 μM) for 24 h. To validate the Cis‐resistant phenotype of SCC9‐CisR cells, parental SCC9 cells and SCC9‐CisR cells were treated with the same Cis concentration gradient and compared in parallel. For experiments evaluating Cis sensitivity under different treatments, SCC9‐CisR cells were pretreated with Rha, 2‐DG, LacNa, or the indicated combinations before exposure to Cis. After treatment, 10 μL of CCK‐8 reagent (catalog number C0037; Beyotime) was added to each well, followed by incubation for 2 h. Absorbance at 450 nm was measured using the Infinite 200 PRO microplate reader (TECAN, Switzerland). Cell viability was plotted against Cis concentration to generate a dose–response curve, from which the half‐maximal inhibitory concentration (IC_50_) was calculated.

To evaluate the cytotoxicity of Rha, normal oral epithelial cells, SCC9 cells, and SCC9‐CisR cells were treated with Rha at 0, 5, 10, or 20 μM for 24 h. Cell viability was then measured using the same CCK‐8 procedure described above and calculated relative to the vehicle‐treated control group.

### 2.9 Reverse Transcription‐Quantitative Polymerase Chain Reaction (RT‐qPCR)

Cultured cells were processed for total RNA isolation using the Yeasen kit according to the manufacturer's protocol (catalog number 19221ES; Yeasen). After extraction, RNA purity was verified (A260/A280: 1.8–2.0) using a Thermo UV spectrophotometer. For reverse transcription, 1 μg of RNA was converted to cDNA using the Hifair AdvanceFast 1st Strand cDNA Synthesis Kit (catalog number 11149ES; Yeasen). Quantitative PCR was performed using the Hieff UNICON Universal Blue qPCR SYBR Green Master Mix (catalog number 11184ES; Yeasen). Gene expression was quantified using the 2^−ΔΔCq^ method [18]. The following primers were used: HIF‐1α forward, 5ˊ‐GAACGTCGAAAAGAAAAGTCTCG‐3′; HIF‐1α reverse, 5ˊ‐CCTTATCAAGATGCGAACTCACA‐3′; β‐actin forward, 5ˊ‐CACCATTGGCAATGAGCGGTTC‐3′; and β‐actin reverse, 5ˊ‐AGGTCTTTGCGGATGTCCACGT‐3′.

### 2.10 Subcutaneous Xenograft Assay

All animal studies were conducted according to the guidelines approved by the Institutional Review Board of Jiangxi University of Chinese Medicine. Male BALB/c nude mice (4 weeks old, 20–25 g), purchased from Nanchang University Animal Center (Nanchang, China), were maintained in a specific pathogen‐free facility. SCC9‐CisR cells (5 × 10^5^ in PBS) were subcutaneously injected into nude mice. When the tumor volume reached approximately 100 mm^3^, the mice were randomized and treated with (1) vehicle control, (2) Cis alone, (3) Rha alone, or (4) Cis in combination with Rha (*n* = 6). Mice were gavaged once daily with Cis (4 mg/kg) alone, Rha (20 mg/kg) alone, or their combination. Control animals received matching volumes of the solvent carrier (1% DMSO in saline). Tumor size was measured every 3 days using the following formula: tumor volume = 0.5 × length × width^2^ (mm^3^). On day 31, the animals were sacrificed, and the tumor tissues were harvested and weighed.

### 2.11 Liver Metastasis Assay

Liver metastases were induced in BALB/c mice by intrasplenic inoculation of SCC9 cells (5 × 10^5^). The animals then received daily gavage of Rha (20 mg/kg), while control animals received an equal volume of vehicle (1% DMSO in saline) (*n* = 6). Three weeks later, the livers were dissected, and the metastatic burden was quantified by counting the nodules.

### 2.12 Western Blot

Cultured cells and xenograft tumor tissues were lysed in RIPA buffer (catalog number P0013B; Beyotime) to obtain total protein extracts. After protein concentration was determined using a BCA assay kit (catalog number P0010; Beyotime), the samples were denatured and subjected to SDS‐PAGE for immunoblotting analysis, followed by transfer to polyvinylidene fluoride membranes. After blocking with QuickBlock buffer (catalog number P0228; Beyotime), the membranes were incubated overnight at 4°C with primary antibodies (Table S1) and then for 2 h with the appropriate secondary antibodies. Protein bands were visualized using an enhanced chemiluminescence detection system (catalog number P0018; Beyotime) according to the standard protocol.

### 2.13 Cycloheximide (CHX) Chase Assay

To evaluate the effect of Rha on HIF‐1α protein stability, a CHX chase assay was performed. SCC9‐CisR cells were pretreated with vehicle or Rha (10 μM) for 24 h, followed by exposure to CHX (50 μg/mL) to block de novo protein synthesis. Cells were collected at the indicated time points after CHX treatment, and HIF‐1α protein levels were examined by western blotting. β‐actin was used as the loading control. The relative HIF‐1α protein level at 0 h was set as 1, and protein degradation curves were generated based on densitometric quantification from three independent experiments.

### 2.14 Chromatin Immunoprecipitation qPCR (ChIP‐qPCR)

ChIP‐qPCR was performed to examine H3K18la enrichment at the promoter regions of glycolysis‐related genes. Briefly, SCC9‐CisR cells were treated with vehicle or Rha (10 μM) for 24 h. Cells were then crosslinked with 1% formaldehyde for 10 min at room temperature, and the reaction was quenched with 125 mM glycine for 5 min. After washing with cold PBS, the cells were harvested and lysed, and chromatin was sheared by sonication to obtain DNA fragments of approximately 200–500 bp. A fraction of the sheared chromatin was reserved as input, and the remaining chromatin was immunoprecipitated overnight at 4°C using an anti‐H3K18la antibody or normal IgG. Protein A/G magnetic beads were then added to capture the immune complexes. After sequential washing, the chromatin complexes were eluted and reverse‐crosslinked at 65°C overnight. DNA was purified and subjected to qPCR using primers targeting the promoter regions of HK2 (forward: 5ˊ‐AAAAAGCTTTGCCAGTTTCCACTG TGTGT‐3′; reverse: 5ˊ‐AAACTCGAGAAGCAGATGCGAGGCAATCA‐3′), LDHA (forward: 5ˊ‐CTTCTGCACACCTCTTCCCA‐3′; reverse: 5ˊ‐TCTCACCTCAAACACACAGCT‐3′), and SLC2A1 (forward: 5ˊ‐AACGAAAACAGCCTCACTGG‐3′; reverse: 5ˊ‐CTGGGACGCCTTCCTCTACT‐3′). H3K18la enrichment was calculated as the percentage of input. Normal IgG served as a negative control. All experiments were performed in triplicate.

### 2.15 Statistical Analysis

Data are reported as the mean ± standard deviation (SD). Statistical evaluations for two‐group comparisons were performed using a two‐tailed Student's *t*‐test, whereas comparisons involving three or more groups were conducted using one‐way or two‐way analysis of variance (ANOVA), as applicable. GraphPad Prism software (GraphPad Software Inc., La Jolla, CA, USA) was used for statistical analyses, with *p* < 0.05 established as the criterion for statistical significance.

## 3 Results

### 3.1 Rha Represses Glycolysis and Histone Lactylation in TSCC Cells

Since SCC9‐CisR cells served as the Cis‐resistant model in this study, their resistant phenotype was first validated. CCK‐8 analysis showed that SCC9‐CisR cells exhibited a right‐shifted Cis dose–response curve compared with parental SCC9 cells, with a significantly higher IC50 value (Figure S1A). These results confirmed that SCC9‐CisR cells retained a stable Cis‐resistant phenotype. We also assessed the cytotoxicity of Rha in normal oral epithelial cells and TSCC cells. At 5 and 10 μM, Rha caused minimal viability loss in normal oral epithelial cells and only modestly affected SCC9 and SCC9‐CisR cells, whereas 20 μM Rha produced a more pronounced reduction in cell viability (Figure S1B). These data indicate that the working concentration of 10 μM had limited nonspecific cytotoxicity.

**FIGURE 1:**
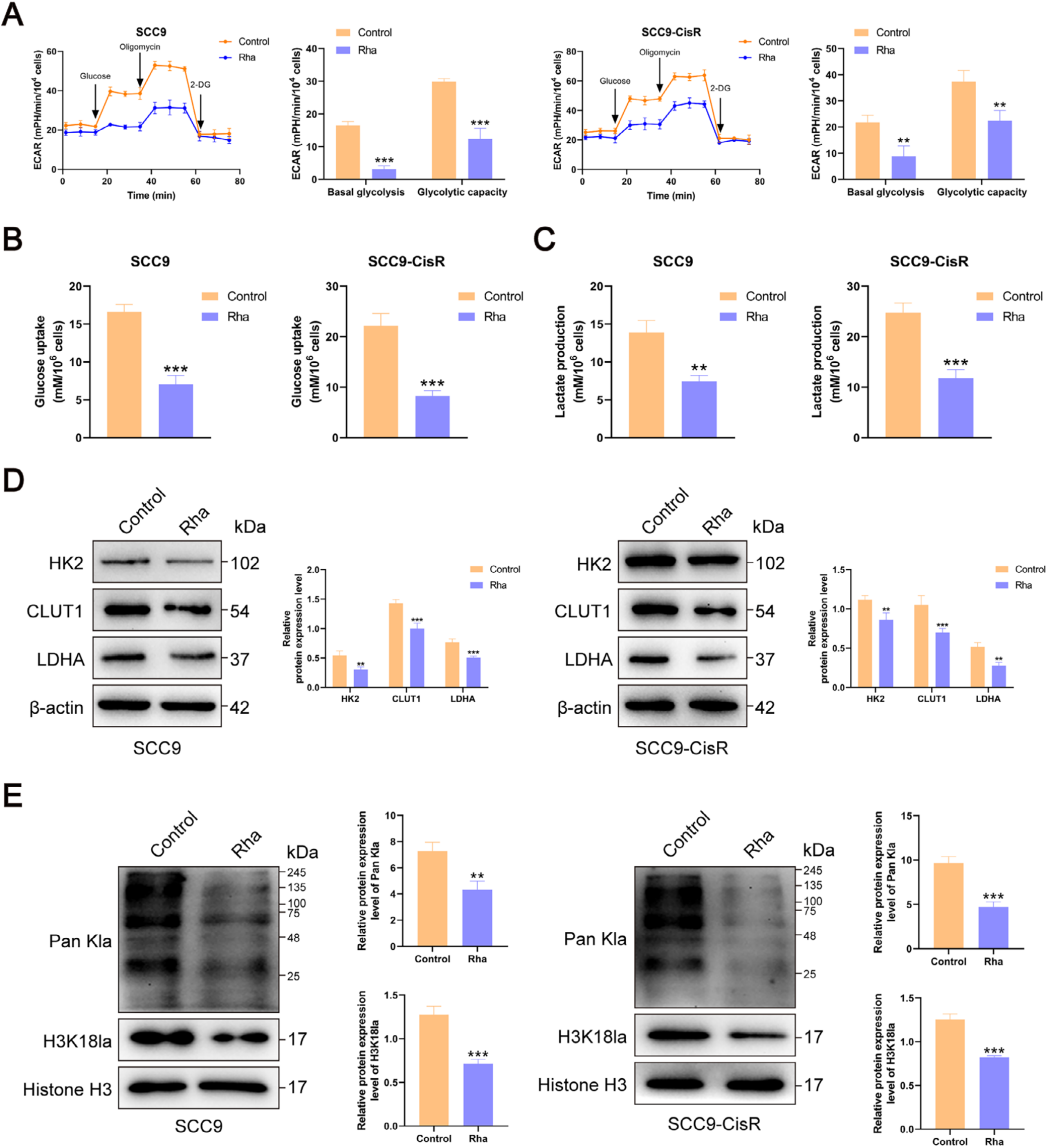
Rha suppresses both glycolysis and histone lactylation in TSCC cells. (A) Basal glycolysis and glycolytic capacity were measured by ECAR in SCC9 and SCC9‐CisR cells treated with or without Rha (*n* = 3). (B, C) Glucose consumption and lactate production in SCC9 and SCC9‐CisR cells were quantified using commercial kits (*n* = 3). (D) Western blotting was used to detect the expression levels of HK2, GLUT1, and LDHA in SCC9 and SCC9‐CisR cells (*n* = 3). (E) The levels of Pan‐Kla and H3K18la in SCC9 and SCC9‐CisR cells were measured by western blotting (*n* = 3). Data are presented as the mean ± SD, and statistical significance was determined by unpaired *t*‐test (B, C, E) and two‐way ANOVA (A, D), **p < 0.01 and ***p < 0.001.

We next explored the impact of Rha on glycolysis and histone lactylation in parental SCC9 cells and their Cis‐resistant counterparts. Measurements of ECAR revealed that Rha treatment significantly suppressed both the basal glycolysis and glycolytic capacity in these two cell lines relative to the control group (Figure 1A). Moreover, a significant reduction in glucose consumption and lactate production was observed in these two cell lines after Rha treatment (Figure 1B,C). Furthermore, Rha treatment led to the downregulation of key glycolytic enzymes, including HK2, LDHA, and GLUT1 (Figure 1D). Building on the finding that lactate serves as a precursor for histone lysine lactylation to regulate gene expression [19], we next examined the impact of Rha on lysine lactylation in both cell lines. The results showed that Rha treatment led to a marked reduction in the protein levels of Pan Kla and H3K18la in these two cell lines (Figure 1E). These findings manifested that Rha inhibits glycolysis and reduces histone lactylation in TSCC cells.

### 3.2 Rha Exerts an Anti‐Tumor Role in TSCC by Inhibiting the Glycolysis‐Lactate Pathway

**FIGURE 2:**
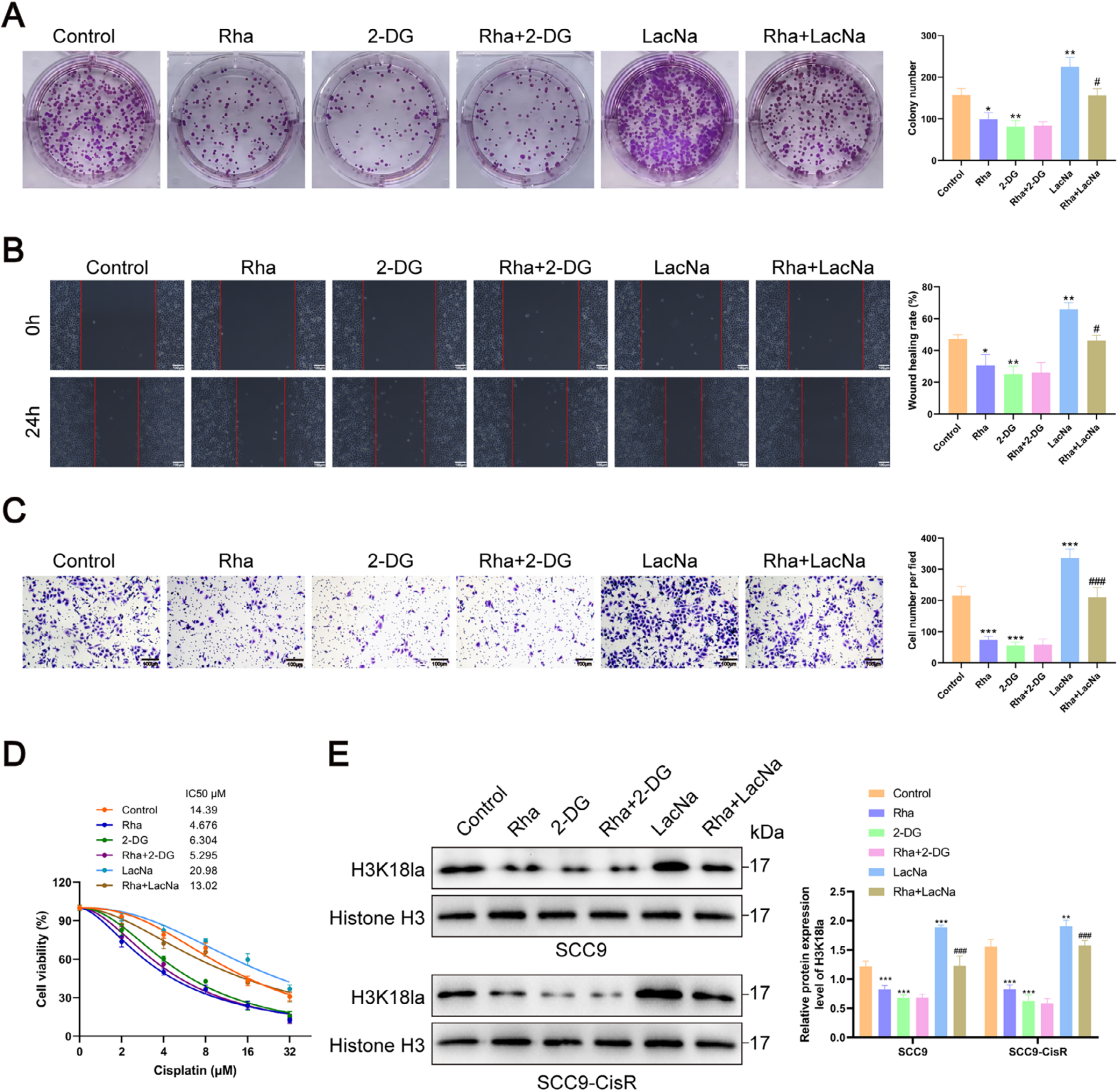
Rha suppresses TSCC malignancy by modulating glycolysis‐derived lactate and H3K18la levels. (A–C) Colony formation, wound‐healing, and Transwell invasion assays were used to evaluate the clonogenicity, migration, and invasion of SCC9 cells treated with control, Rha, 2‐DG, Rha + 2‐DG, LacNa, or Rha + LacNa (*n* = 3). (D) The IC_50_ of Cis for SCC9‐CisR cells treated with control, Rha, 2‐DG, Rha + 2‐DG, LacNa, or Rha + LacNa was determined using the CCK‐8 assay (*n* = 3). (E) The protein levels of H3K18la in the aforementioned SCC9 and SCC9‐CisR cells were analyzed by western blotting (*n* = 3). Data are presented as the mean ± SD, and statistical significance was determined by one‐way ANOVA (A‐C) and two‐way ANOVA (D, E), *p < 0.05, **p < 0.01, and ***p < 0.001 versus control; ^#^ *p* < 0.05 and ^###^ *p* < 0.001 versus LacNa.

To investigate whether Rha suppresses TSCC malignancy by modulating glycolysis‐derived lactate and H3K18la levels, we treated SCC9 and SCC9‐CisR cells with Rha alone or in combination with 2‐DG or LacNa (5 mM). Both 2‐DG and Rha inhibited the clonogenic, migratory, and invasive abilities of SCC9 cells. However, the combination of Rha with 2‐DG showed no additive effect compared with 2‐DG treatment alone (Figure 2A–C). While LacNa promoted the clonogenicity, migration, and invasion of SCC9 cells, this promotional effect on malignant behaviors was suppressed by Rha treatment (Figure 2A–C). Although both 2‐DG and Rha individually lowered the IC_50_ of Cis in SCC9‐CisR cells, the Rha and 2‐DG combination provided no additional benefit. Conversely, the elevated IC_50_ value induced by LacNa was effectively counteracted by Rha treatment (Figure 2D). Similarly, both 2‐DG and Rha suppressed H3K18la protein levels in these two cell lines, whereas the combination of Rha with 2‐DG showed no additive effect compared with 2‐DG alone. Conversely, the LacNa‐induced upregulation of H3K18la was effectively reversed by Rha treatment (Figure 2E). Collectively, these results suggest that Rha‐mediated suppression of TSCC malignancy occurs through the modulation of glycolysis‐derived lactate and H3K18la levels.

### 3.3 Rha Regulates Glycolysis‐Derived Lactate and H3K18la via HIF‐1α

Since our previous study confirmed that Rha regulates TSCC cell stemness and Cis sensitivity via the HIF‐1α/MCT4 axis [16], we further investigated whether Rha modulates glycolysis‐derived lactate and H3K18la through HIF‐1α. To clarify whether Rha affects HIF‐1α protein stability, a CHX chase assay was first performed in SCC9‐CisR cells. After blockade of de *novo* protein synthesis by CHX, HIF‐1α protein levels gradually declined in control cells. Notably, Rha‐treated cells exhibited a more rapid decrease in HIF‐1α protein levels than control cells, indicating that Rha shortened the half‐life of HIF‐1α and reduced its protein stability (Figure S2A,B).

**FIGURE 3:**
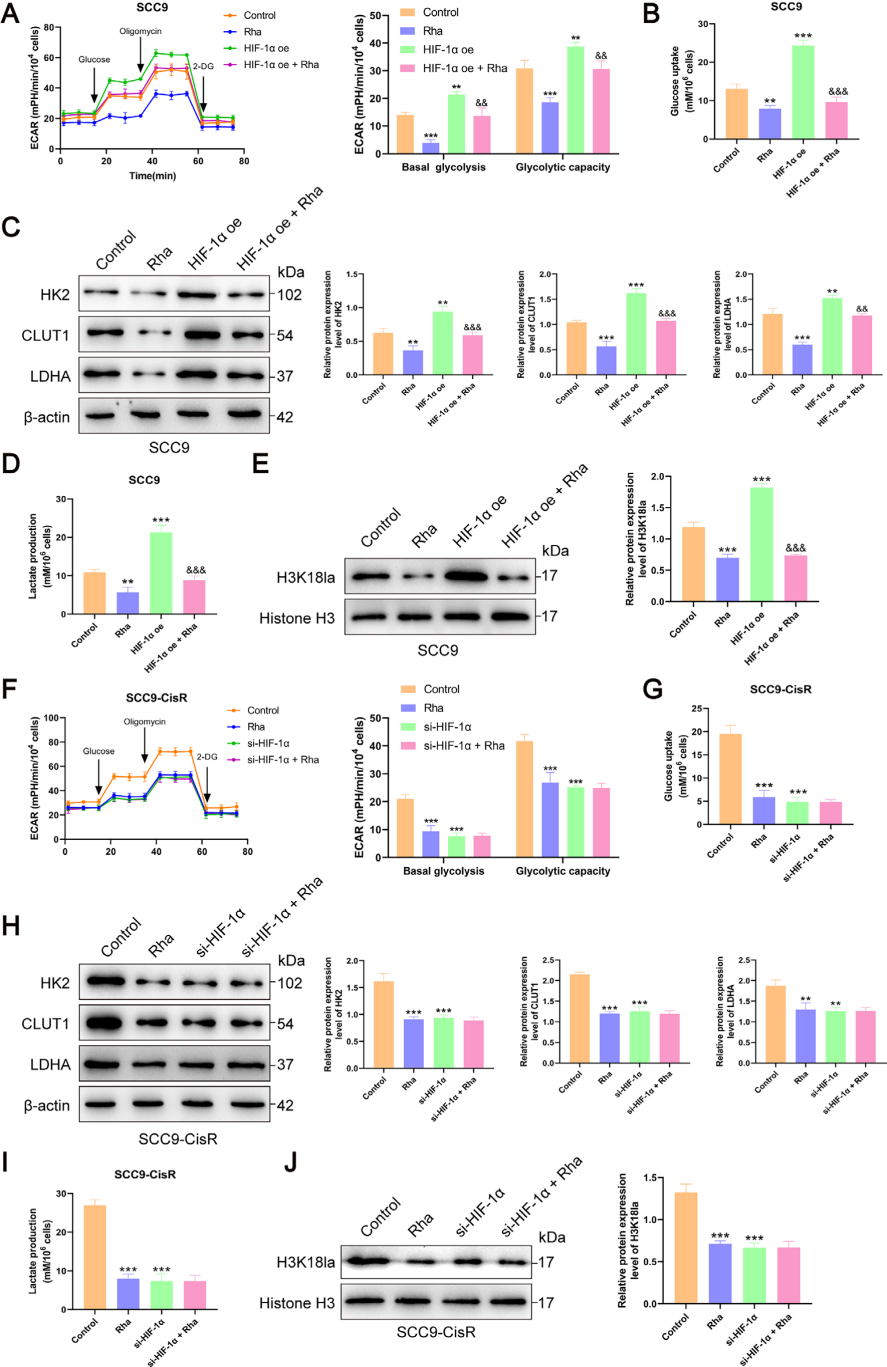
The ability of Rha to regulate glycolysis‐derived lactate and H3K18la is dependent on HIF‐1α. (A) Basal glycolysis and glycolytic capacity were measured in SCC9 cells treated with control, Rha, HIF‐1α oe, or HIF‐1α oe + Rha (*n* = 3). (B‐C) Quantification of glucose consumption and lactate production in SCC9 cells (*n* = 3). (D‐E) Analysis of HK2, LDHA, GLUT1, and H3K18la protein levels in SCC9 cells (*n* = 3). (F) Basal glycolysis and glycolytic capacity were measured in SCC9‐CisR cells treated with control, Rha, si‐HIF‐1α, or si‐HIF‐1α + Rha (*n* = 3). (G‐H) Glucose consumption and lactate production in SCC9‐CisR cells were quantified (*n* = 3). (I) Assessment of HK2, LDHA, GLUT1, and H3K18la protein levels in SCC9‐CisR cells (*n* = 3). Data are presented as the mean ± SD, and statistical significance was determined by one‐way ANOVA (B‐E and G‐I) and two‐way ANOVA (A, F), **p < 0.01 and ***p < 0.001 versus control; ^&&^ *p* < 0.01 and ^&&&^ *p* < 0.001 versus HIF‐1α oe.

The role of HIF‐1α was investigated through its overexpression in SCC9 cells and knockdown in SCC9‐CisR cells. The effectiveness of these interventions was validated by the expected upregulation or downregulation of HIF‐1α mRNA and protein (Figure S3A–D). We observed that HIF‐1α overexpression enhanced basal glycolysis and glycolytic capacity in SCC9 cells, accompanied by increased glucose consumption and lactate production. These alterations induced by HIF‐1α overexpression were reversed by Rha treatment (Figure 3A–C). Consistently, HIF‐1α overexpression elevated HK2, LDHA, GLUT1, and H3K18la protein levels in SCC9 cells, whereas Rha treatment effectively counteracted these HIF‐1α‐mediated effects (Figure 3D,E). Although Rha treatment, similar to HIF‐1α knockdown, suppressed basal glycolysis/glycolytic capacity, glucose uptake, and lactate output in SCC9‐CisR cells, it lost this efficacy in cells in which HIF‐1α had already been knocked down (Figure 3F–H). Similarly, both Rha and HIF‐1α knockdown reduced the protein levels of HK2, LDHA, GLUT1, and H3K18la in SCC9‐CisR cells, but Rha failed to exert this effect when HIF‐1α was knocked down (Figure 3I). Altogether, these data indicate that the regulation of glycolysis‐derived lactate and H3K18la by Rha is dependent on HIF‐1α.

To further determine whether Rha affects gene‐specific H3K18la deposition at glycolysis‐related loci, ChIP‐qPCR was performed in SCC9‐CisR cells. Compared with the control group, Rha treatment significantly reduced H3K18la enrichment at the promoter regions of HK2, LDHA, and SLC2A1 (Figure S4A–C). In contrast, IgG showed minimal background enrichment. These results indicate that Rha not only decreases global H3K18la levels but also reduces H3K18la deposition at glycolysis‐related gene promoters, providing gene‐specific epigenetic evidence for the suppression of glycolytic gene expression.

### 3.4 Rha Represses Tumor Growth and Glycolysis‐Derived H3K18la in Xenograft Models

**FIGURE 4:**
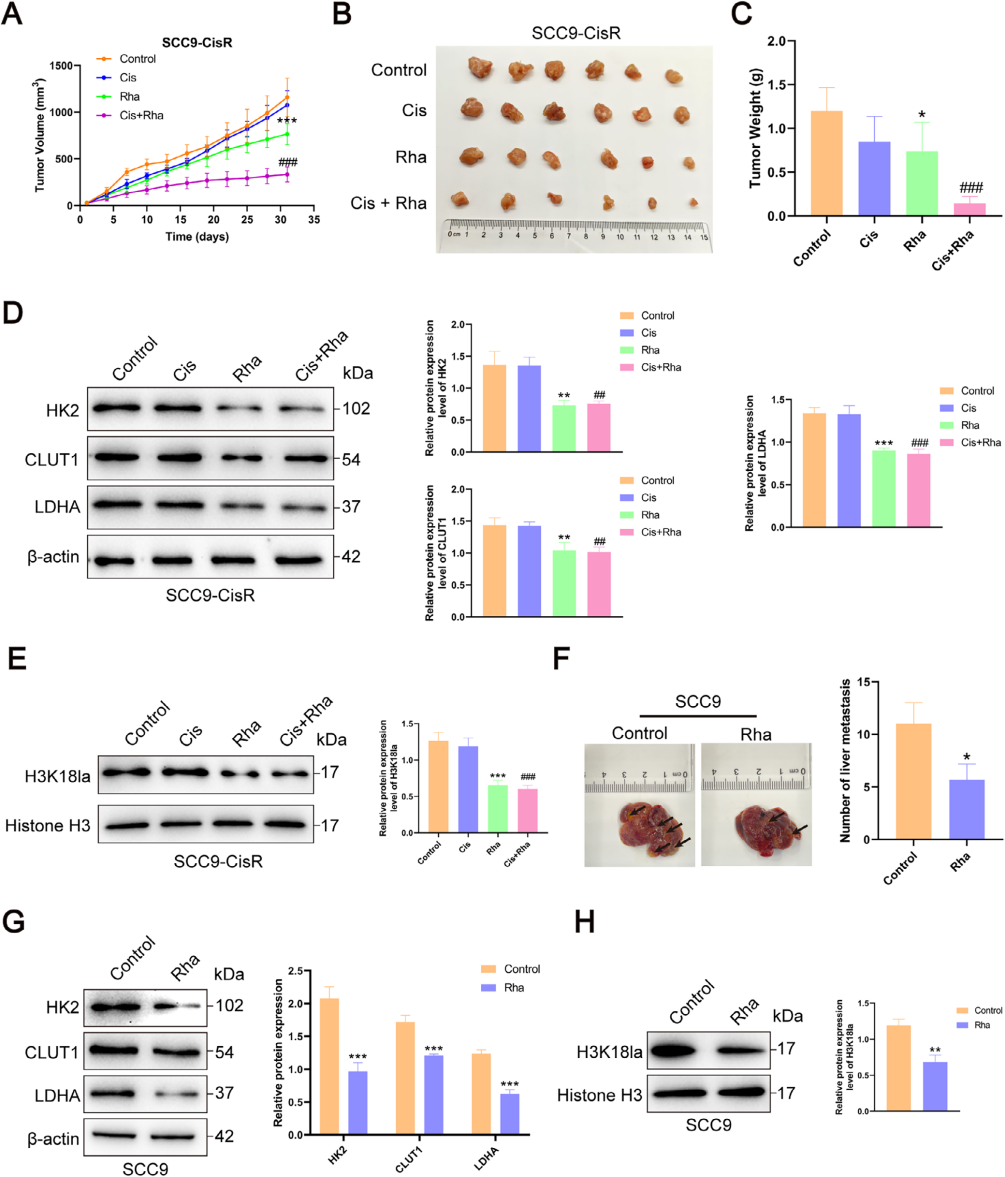
Rha suppresses both tumor growth and glycolysis‐derived H3K18la in TSCC. (A) Tumor growth was monitored by measuring tumor volume (*n* = 6). (B) Representative photograph of xenograft tumors obtained from the different mouse groups. (C) Evaluation of tumor weight across all experimental mouse groups (*n* = 6). (D‐E) Protein levels of HK2, LDHA, GLUT1, and H3K18la were quantified in tumors from each mouse group (*n* = 6). (F) Assessment of liver metastasis in mice following splenic injection of SCC9 cells and treatment with or without Rha (*n* = 6). (G‐H) Protein levels of HK2, LDHA, GLUT1, and H3K18la in tumor‐bearing liver tissues from different groups (*n* = 6). Data are presented as the mean ± SD, and statistical significance was determined by unpaired *t*‐test (F, H), one‐way ANOVA (C‐E), and two‐way ANOVA (A, G), **p < 0.01 and ***p < 0.001 versus control; ^##^ *p* < 0.01 and ^###^ *p* < 0.001 versus Cis.

To validate the in vivo regulation of glycolysis‐derived H3K18la by Rha, a subcutaneous xenograft model was generated using SCC9‐CisR cells, followed by treatment with Cis, Rha, or their combination. The results showed that Rha treatment significantly inhibited tumor growth and reduced tumor weight, whereas Cis showed no significant effect. Interestingly, the combination of Rha and Cis produced a superior anti‐tumor effect compared with either agent alone, suggesting that Rha can ameliorate Cis resistance (Figure 4A–C). Furthermore, Rha, but not Cis, suppressed HK2, LDHA, GLUT1, and H3K18la protein levels in tumors, and their combination produced a greater inhibitory effect than Cis alone (Figure 4D,E). A liver metastasis model was then established through splenic injection of SCC9 cells, followed by treatment with Rha. The results showed that Rha‐treated mice exhibited fewer metastatic nodules in their livers than control mice (Figure 4F). In addition, tumor‐bearing liver tissues from Rha‐treated mice showed lower protein levels of HK2, LDHA, GLUT1, and H3K18la than those from control mice (Figure 4G,H). These results suggest that Rha suppresses tumor growth and metastasis and modulates glycolysis‐derived H3K18la in TSCC.

## 4 Discussion

This study demonstrated that Rha exerts anti‐tumor effects in TSCC through HIF‐1α‐mediated suppression of glycolysis, leading to reduced lactate levels and subsequent downregulation of H3K18la. Modulation of this metabolic‐epigenetic axis offers a promising approach to overcoming cancer treatment resistance.

Metabolic reprogramming constitutes a core hallmark of malignant tumors, in which aerobic glycolysis serves as a critical driver of TSCC invasion and drug resistance [6, 20]. It has been demonstrated that Rha attenuates cancer stemness and potentiates Cis sensitivity in TSCC [16]. The present study showed that Rha exerted potent inhibitory effects on glycolytic metabolism in parental SCC9 cells and their Cis‐resistant counterparts, as evidenced by significantly reduced ECAR, lactate production, and glucose consumption, together with the downregulation of HK2, LDHA, and GLUT1. These findings suggest that Rha curbs glycolysis in TSCC.

Lactylation, a histone post‐translational modification discovered in 2019, establishes a direct link between cellular metabolic status and the regulation of gene transcription [19]. Increased lactate levels drive enhanced histone lactylation, particularly at H3K18. Notably, H3K18la, a specific lactylation mark, has been demonstrated to drive pro‐oncogenic genes in various cancers, including ovarian cancer [21], lung cancer [22], and colorectal cancer [23]. This study demonstrated for the first time that Rha markedly decreased Pan‐Kla and H3K18la levels in parental SCC9 cells and their Cis‐resistant counterparts. Importantly, both Rha and 2‐DG inhibited the clonogenicity, migration, and invasion of SCC9 cells and enhanced Cis sensitivity in SCC9‐CisR cells, accompanied by the downregulation of H3K18la. Conversely, Rha reversed the LacNa‐induced increases in tumor‐promoting effects, Cis resistance, and H3K18la levels. In vivo data confirmed that Rha potently inhibited tumor growth and metastasis and restored Cis sensitivity by targeting the glycolysis/lactate/H3K18la axis, as evidenced by reduced levels of glycolytic enzymes and H3K18la. These results are highly consistent with mechanisms reported in prior studies [24, 25, 26], in which lactate promotes chemotherapy resistance through H3K18la. These data suggest that Rha exerts a tumor‐inhibitory role and enhances Cis sensitivity by targeting glycolysis‐derived lactate and H3K18la levels. Furthermore, the ChIP‐qPCR results provided gene‐specific epigenetic evidence that Rha reduced H3K18la enrichment at the promoter regions of HK2, LDHA, and SLC2A1 in SCC9‐CisR cells. This finding extends the western blot results showing decreased global H3K18la levels and supports the notion that Rha suppresses glycolytic gene expression, at least in part, by attenuating H3K18la deposition at glycolysis‐related promoters. Nevertheless, the current ChIP‐qPCR analysis focused only on selected glycolytic genes. Future studies integrating ChIP‐seq and RNA‐seq will be valuable for comprehensively defining the genome‐wide H3K18la landscape and the full transcriptional programs regulated by Rha in TSCC.

HIF‐1α, a central transcription factor for glycolytic metabolism, drives the expression of glycolytic enzymes [27]. Using genetic gain‐ and loss‐of‐function approaches, we established that Rha inhibited glycolysis strictly via HIF‐1α. In addition to confirming the dependence of Rha‐mediated glycolytic suppression on HIF‐1α, the present study further examined whether Rha affects HIF‐1α protein stability. The CHX chase assay showed that Rha accelerated the decline of HIF‐1α protein after blockade of de novo protein synthesis, indicating that Rha shortened the half‐life of HIF‐1α. These findings extend our previous observations and suggest that Rha suppresses the HIF‐1α‐driven glycolysis/lactate/H3K18la axis, at least in part, by reducing HIF‐1α protein stability. However, the precise degradation machinery involved in this process requires further investigation. This finding represents a logical extension of our previous report showing that Rha regulates TSCC stemness and Cis resistance via the HIF‐1α/MCT4 axis [16]. Zhang et al. demonstrated that H3K18la is a key epigenetic regulator of hypoxia‐induced colon cancer stem cell properties [28]. Dong et al. found that HIF‐1α‐driven H3K18la modification activates a mitophagy‐metabolic reprogramming axis, thereby promoting glioma malignancy [29]. These results indicate that Rha exerts anti‐tumor activity in TSCC by inhibiting the glycolysis/lactate/H3K18la axis through its regulatory effect on HIF‐1α.

Drug safety is an important consideration for the development of Rha‐based therapeutic strategies. Our cell viability assay showed that Rha had little effect on normal oral epithelial cells at 5 and 10 μM and only modestly affected SCC9 and SCC9‐CisR cells at the working concentration of 10 μM. These data suggest limited nonspecific cytotoxicity under the present experimental conditions. However, this in vitro evidence remains preliminary, and further pharmacokinetic, toxicological, and long‐term in vivo safety studies are needed before clinical translation.

Although the present study provided promoter‐level evidence that Rha reduced H3K18la enrichment at selected glycolysis‐related genes, the analysis was limited to HK2, LDHA, and SLC2A1. Genome‐wide ChIP‐seq combined with RNA‐seq will be required in future studies to comprehensively identify the broader H3K18la‐dependent transcriptional network regulated by Rha in TSCC.

In summary, this study revealed that Rha inhibits the glycolysis/lactate/H3K18la axis in a HIF‐1α‐dependent manner, thereby effectively suppressing TSCC progression and overcoming Cis resistance. These findings provide important experimental evidence and a novel rationale for developing Rha‐based or pathway‐targeted therapeutic strategies against TSCC, particularly for Cis‐resistant patients.

## Funding

This work was supported by the National Natural Science Foundation of China (grant number 82460954); and Finance and Administration Department of the Health Commission of Jiangxi Province (grant number 52526803).

## Conflicts of Interest

The authors declare no conflicts of interest.

## Supporting information


**Figure S1:** Validation of the Cis‐resistant phenotype of SCC9‐CisR cells. (A) SCC9 and SCC9‐CisR cells were treated with increasing concentrations of Cis for 24 h, and cell viability was measured using the CCK‐8 assay. Dose–response survival curves were generated to compare Cis sensitivity between parental SCC9 cells and SCC9‐CisR cells. (B) Normal oral epithelial cells, SCC9 cells, and SCC9‐CisR cells were treated with Rha at 0, 5, 10, or 20 μM for 24 h, and cell viability was assessed using the CCK‐8 assay. Data are presented as the mean ± SD from three independent experiments. Statistical significance was determined by two‐way ANOVA (B). *p < 0.05, **p < 0.01 and, ***p < 0.001 vs. control.

**Figure S2:** Rha decreases HIF‐1α protein stability in SCC9‐CisR cells. (A) SCC9‐CisR cells were pretreated with vehicle or Rha (10 μM) for 24 h and then exposed to CHX (50 μg/mL). HIF‐1α protein levels were detected by western blotting at the indicated time points. (B) Quantification of HIF‐1α protein stability after CHX treatment. The HIF‐1α level at 0 h was set as 100%. Data are presented as the mean ± SD from three independent experiments.

**Figure S3:** Assessment of HIF‐1α overexpression and knockdown efficiency. (A‐B) Relative mRNA and protein levels of HIF‐1α in SCC9 cells transfected with vector or HIF‐1α oe were detected by RT‐qPCR and western blotting (*n* = 3). (C‐D) Relative mRNA and protein levels of HIF‐1α in SCC9‐CisR cells transfected with si‐NC, si‐HIF‐1α#1, or si‐HIF‐1α#2 were detected by RT‐qPCR and western blotting (*n* = 3). Data are presented as the mean ± SD, and statistical significance was determined by one‐way ANOVA (A‐D), **p < 0.01 and ***p < 0.001 vs. control.

**Figure S4:** Rha reduces H3K18la enrichment at glycolysis‐related gene promoters in SCC9‐CisR cells. SCC9‐CisR cells were treated with vehicle or Rha (10 μM) for 24 h, followed by ChIP‐qPCR using an anti‐H3K18la antibody or normal IgG. H3K18la enrichment at the promoter regions of (A) HK2, (B) LDHA, and (C) SLC2A1 was quantified and expressed as a percentage of input. IgG served as a negative control and showed minimal enrichment. Data are presented as the mean ± SD from three independent experiments. Statistical significance was determined by an unpaired Student's *t*‐test between the Control‐H3K18la and Rha‐H3K18la groups (A‐C). ***p < 0.001 vs. Control‐H3K18la.

**Table S1:** The antibodies used in this research.

## Data Availability

The data that support the findings of this study are available from the corresponding author upon reasonable request.
