# Supplementary figures and images for: Rhaponticin Blocks Glycolysis‐Mediated Histone Lactylation to Suppress Tongue Squamous Cell Carcinoma via HIF‐1α Activity Inhibition
Source: Kaohsiung J Med Sci. 2026 Jun 18:e70254. Online ahead of print. doi: 10.1002/kjm2.70254 (PMC13399672; doi:10.1002/kjm2.70254)

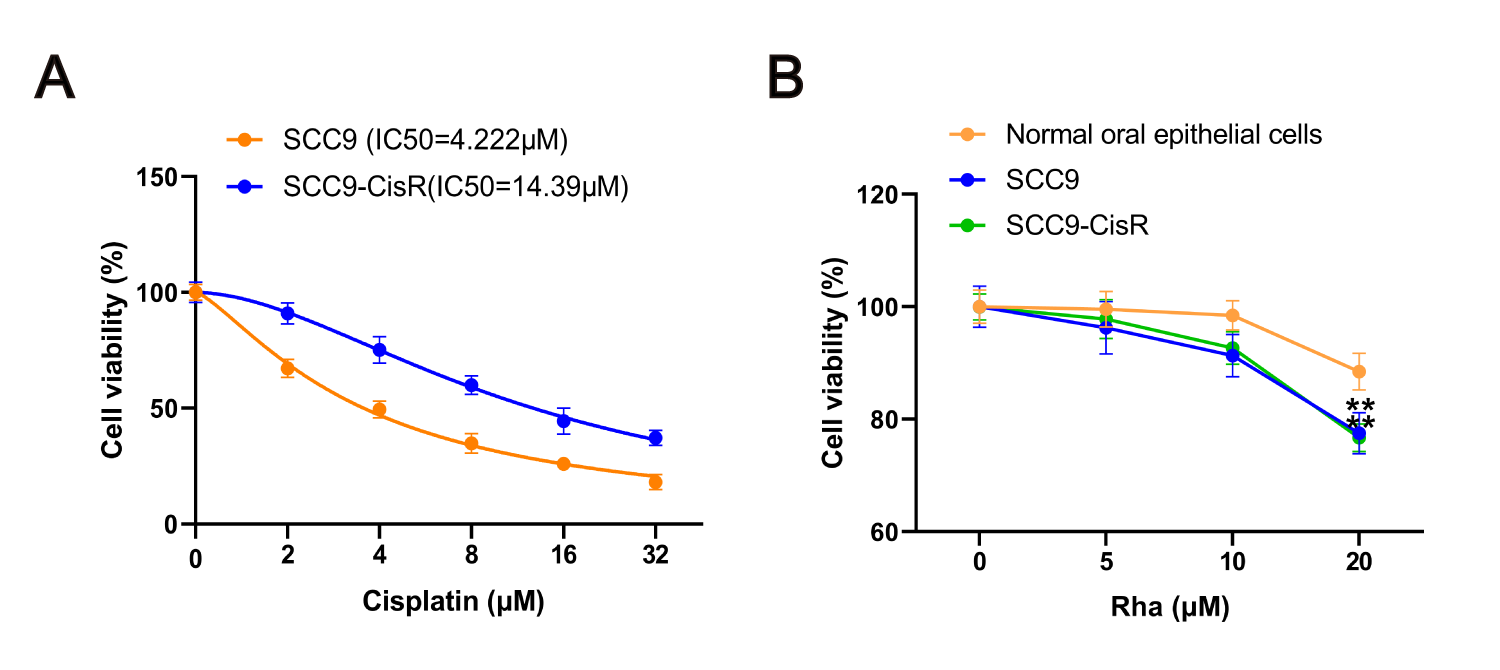

Supplement: Supplementary file 1 — Figure S1: Validation of the Cis‐resistant phenotype of SCC9‐CisR cells. (A) SCC9 and SCC9‐CisR cells were treated with increasing concentrations of Cis for 24 h, and cell viability was measured using the CCK‐8 assay. Dose–response survival curves were generated to compare Cis sensitivity between parental SCC9 cells and SCC9‐CisR cells. (B) Normal oral epithelial cells, SCC9 cells, and SCC9‐CisR cells were treated with Rha at 0, 5, 10, or 20 μM for 24 h, and cell viability was assessed using the CCK‐8 assay. Data are presented as the mean ± SD from three independent experiments. Statistical significance was determined by two‐way ANOVA (B). *p < 0.05, **p < 0.01 and, ***p < 0.001 vs. control. [file KJM2-9999-e70254-s005.tif]

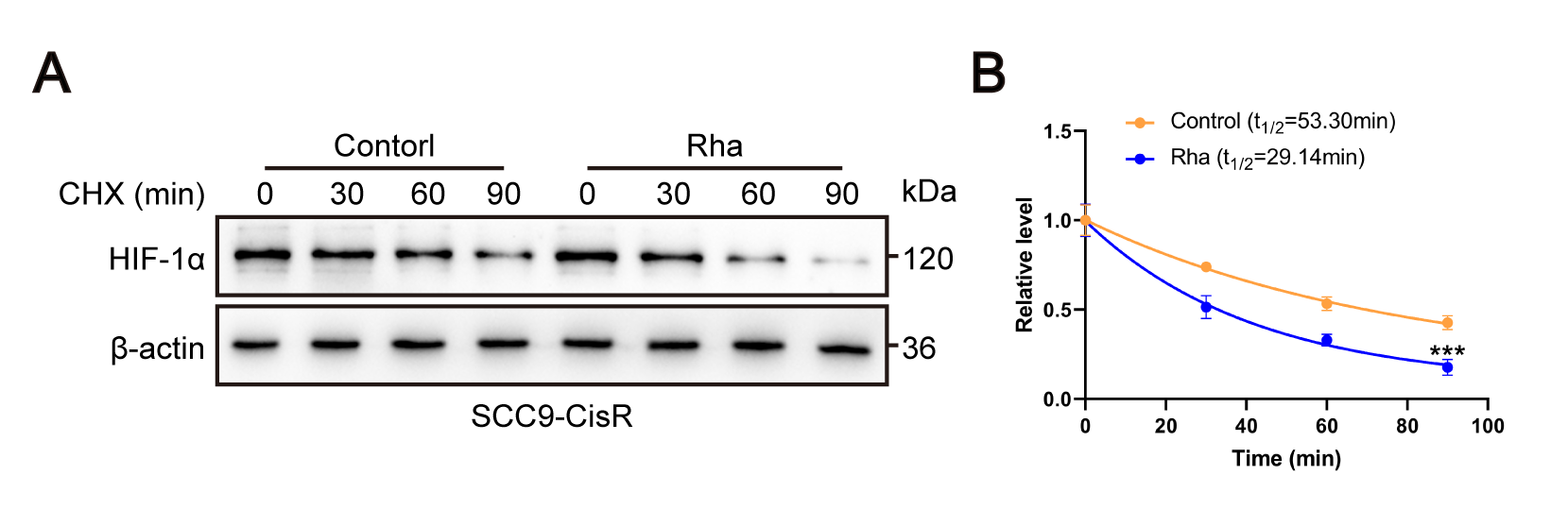

Supplement: Supplementary file 2 — Figure S2: Rha decreases HIF‐1α protein stability in SCC9‐CisR cells. (A) SCC9‐CisR cells were pretreated with vehicle or Rha (10 μM) for 24 h and then exposed to CHX (50 μg/mL). HIF‐1α protein levels were detected by western blotting at the indicated time points. (B) Quantification of HIF‐1α protein stability after CHX treatment. The HIF‐1α level at 0 h was set as 100%. Data are presented as the mean ± SD from three independent experiments. [file KJM2-9999-e70254-s001.tif]

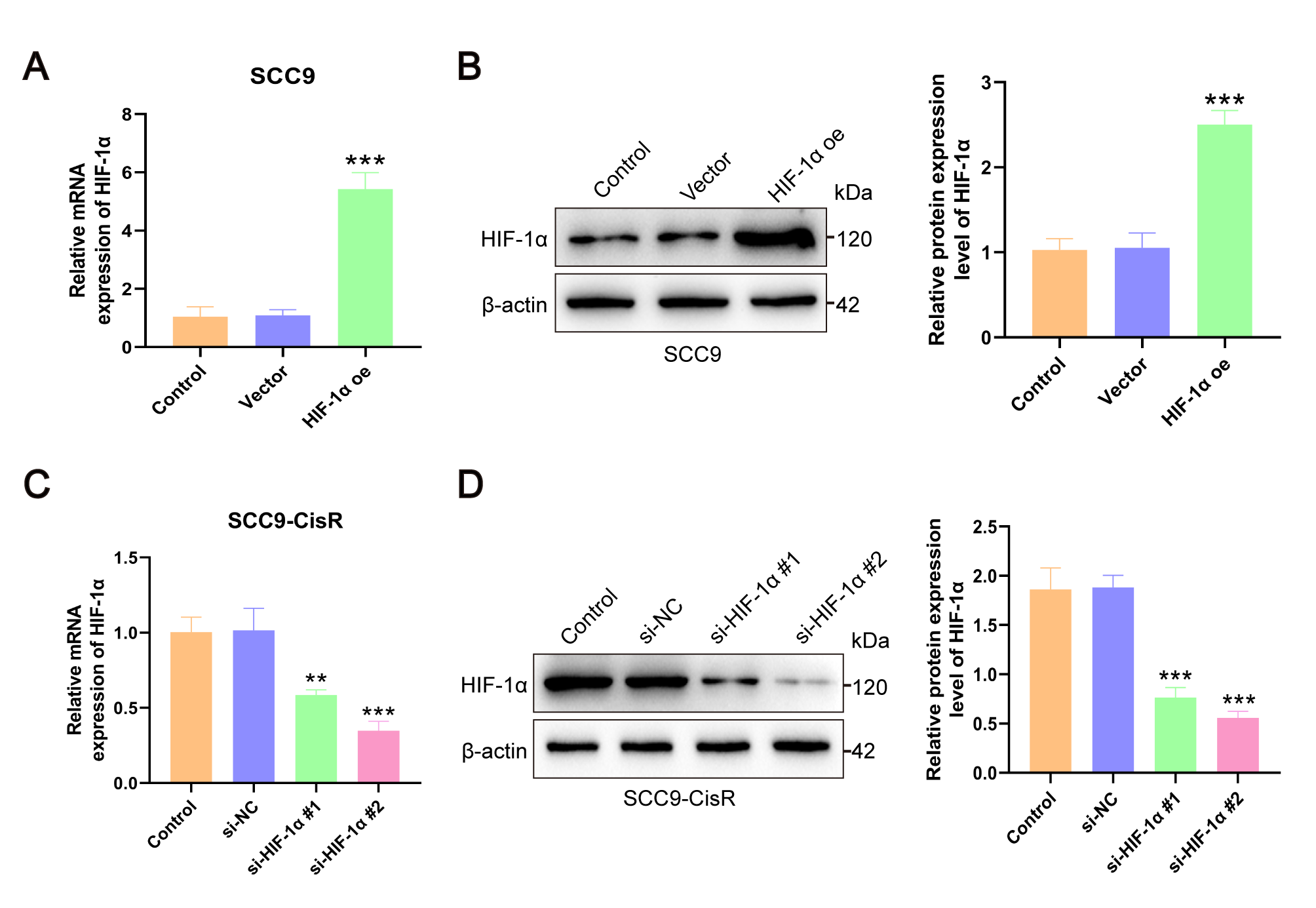

Supplement: Supplementary file 3 — Figure S3: Assessment of HIF‐1α overexpression and knockdown efficiency. (A‐B) Relative mRNA and protein levels of HIF‐1α in SCC9 cells transfected with vector or HIF‐1α oe were detected by RT‐qPCR and western blotting (n = 3). (C‐D) Relative mRNA and protein levels of HIF‐1α in SCC9‐CisR cells transfected with si‐NC, si‐HIF‐1α#1, or si‐HIF‐1α#2 were detected by RT‐qPCR and western blotting (n = 3). Data are presented as the mean ± SD, and statistical significance was determined by one‐way ANOVA (A‐D), **p < 0.01 and ***p < 0.001 vs. control. [file KJM2-9999-e70254-s004.tif]

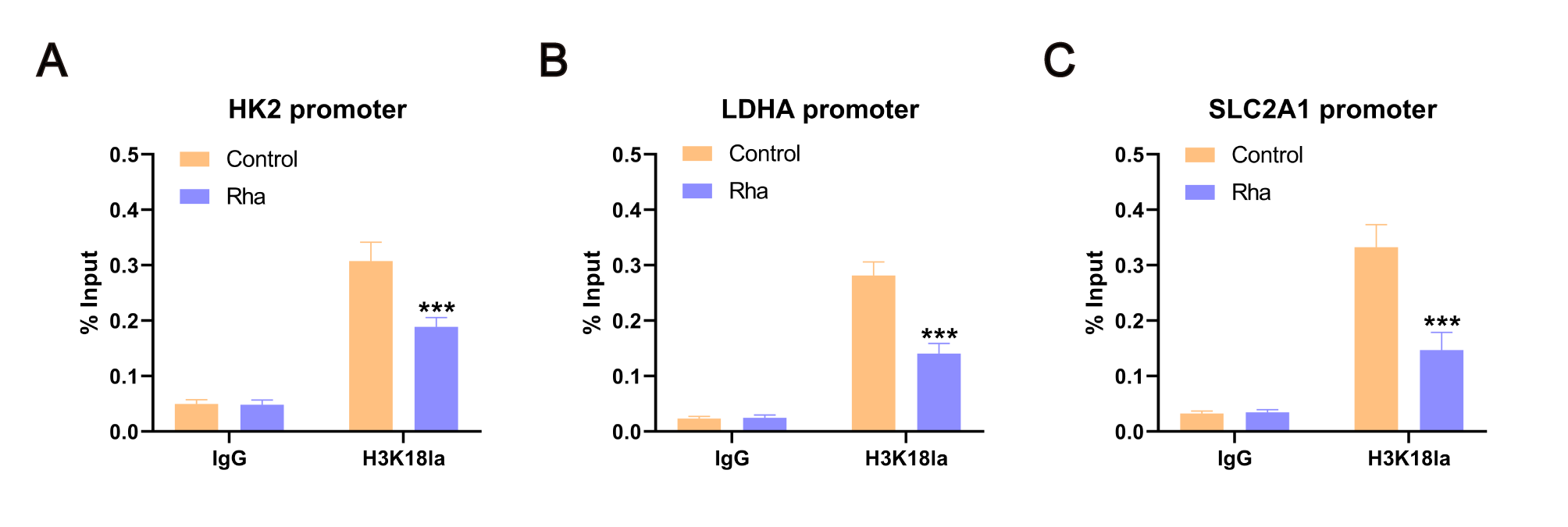

Supplement: Supplementary file 4 — Figure S4: Rha reduces H3K18la enrichment at glycolysis‐related gene promoters in SCC9‐CisR cells. SCC9‐CisR cells were treated with vehicle or Rha (10 μM) for 24 h, followed by ChIP‐qPCR using an anti‐H3K18la antibody or normal IgG. H3K18la enrichment at the promoter regions of (A) HK2, (B) LDHA, and (C) SLC2A1 was quantified and expressed as a percentage of input. IgG served as a negative control and showed minimal enrichment. Data are presented as the mean ± SD from three independent experiments. Statistical significance was determined by an unpaired Student's t‐test between the Control‐H3K18la and Rha‐H3K18la groups (A‐C). ***p < 0.001 vs. Control‐H3K18la. [file KJM2-9999-e70254-s002.tif]
