# Supplementary material for: Rhaponticin Blocks Glycolysis‐Mediated Histone Lactylation to Suppress Tongue Squamous Cell Carcinoma via HIF‐1α Activity Inhibition
Source: Kaohsiung J Med Sci. 2026 Jun 18:e70254. Online ahead of print. doi: 10.1002/kjm2.70254 (PMC13399672; doi:10.1002/kjm2.70254)
Supplement: Supplementary file 5 — Table S1: The antibodies used in this research. [file KJM2-9999-e70254-s003.docx]

**Table S1 The antibodies used in this research**

| **Antibody** | **Manufacturer** | **Cat.no** | **Dilution** |
| --- | --- | --- | --- |
| HK2 | Cell Signaling | 2867 | 1:1,000 |
| GLUT1 | ABclonal | A11727 | 1:1,000 |
| LDHA | ABclonal | A21893 | 1:1,000 |
| Pan Kla | ABclonal | A23004 | 1:1,000 |
| H3K18la | ABclonal | A18807 | 1:1,000 |
| HIF-1α | Beyotime | AH339 | 1:500 |
| Histone H3 | Beyotime | AF0009 | 1:1,000 |
| β-actin | Beyotime | AF5003 | 1:1,000 |
| HRP conjugated Goat Anti-Mouse IgG (H+L) | Beyotime | A0216 | 1:1,000 |
| HRP conjugated Goat Anti-Rabbit IgG (H+L) | Beyotime | A0208 | 1:1,000 |
